# Ketamine in Diabetes Care: Metabolic Insights and Clinical Applications

**DOI:** 10.3390/pharmaceutics18010081

**Published:** 2026-01-08

**Authors:** Shiryn D. Sukhram, Majandra Sanchez, Ayotunde Anidugbe, Bora Kupa, Vincent P. Edwards, Muhammad Zia, Grozdena Yilmaz

**Affiliations:** 1 Department of Biology, College of Staten Island, New York, NY 10314, USA; bora.kupa@cix.csi.cuny.edu (B.K.); muhammad.zia@csi.cuny.edu (M.Z.); grozdena.yilmaz@csi.cuny.edu (G.Y.); 2 College of Pharmacy, Long Island University, New York, NY 11201, USA; majandra.sanchez@my.liu.edu (M.S.); ayotunde.anidugbe@my.liu.edu (A.A.); 3 College of Arts and Sciences, Seton Hall University, South Orange, NJ 07079, USA; vincent.edwards@student.shu.edu

**Keywords:** population pharmacokinetics, PK-PD, ketamine, esketamine, diabetes mellitus, diabetic neuropathy, model-informed precision dosing, exposure–response, CYP2B6, CYP3A4

## Abstract

**Background:** Depression and diabetic neuropathy (DN) commonly complicate diabetes and impair glycemic control and quality of life. Ketamine and its S-enantiomer, esketamine, provide rapid antidepressant and analgesic effects, yet diabetes-related pathophysiology and co-therapies may modify exposure, response, and safety. **Methods:** We conducted a scoping review following PRISMA-ScR. MEDLINE/PubMed, CINAHL, and APA PsycInfo were searched (January 2020–31 May 2025). Eligible human and animal studies evaluated ketamine, esketamine, or norketamine in the context of diabetes (type 1 [T1DM], type 2 [T2DM], gestational [GDM]), or DN, and reported psychiatric, analgesic, metabolic, or mechanistic outcomes. Two reviewers independently screened and charted data; no formal risk-of-bias assessment was performed. **Results:** Eleven studies met inclusion criteria: four human case reports/series (three T1DM, one T2DM), one randomized trial in GDM, two narrative reviews of topical ketamine for DN, and four preclinical rodent studies using streptozotocin- or diet-induced diabetes models. Short-term improvements were reported for treatment-resistant depression and neuropathic pain, including opioid-sparing postoperative analgesia in GDM. Glycemic effects varied across settings, with both hyperglycemia and hypoglycemia reported. Mechanistic and clinical drug–drug and drug-disease interactions (particularly involving metformin, GLP-1 receptor agonists, SGLT2 inhibitors, and CYP3A4/CYP2B6 modulators) remain insufficiently studied. We outline a forward-looking population pharmacokinetic (popPK) and pharmacokinetic-pharmacodynamic (PK-PD) research agenda, including priority covariates (eGFR, hepatic function, inflammatory status, HbA1c, genotype, co-medications) and sparse-sampling windows for future model-informed precision dosing. **Conclusions:** Current evidence supports cautious, selective use of ketamine for refractory depression and DN within multidisciplinary diabetes care. Purpose-built popPK/PK-PD studies in both human and preclinical diabetic models cohorts are needed to quantify variability, define drug–disease–drug interactions and glycemic risk, and inform individualized dosing strategies.

## 1. Introduction

Diabetes encompasses a group of chronic metabolic disorders characterized by persistent hyperglycemia resulting from impaired insulin secretion, insulin resistance, or both [1]. Despite advances in pharmacotherapy, clinical management remains complex because of multimorbidity and heterogeneous pathophysiology. Depression is one of the most common and disabling psychiatric disorders globally [2] and is especially prevalent among individuals with chronic medical illness; people living with diabetes are at particularly high risk. Up to 30% of individuals with diabetes experience comorbid depression, nearly twice the prevalence observed in the general population [3]. This bidirectional relationship worsens clinical outcomes, increases treatment burden, and contributes to premature morbidity and mortality. Chronic pain and depression frequently co-occur in diabetes and further compromise adherence, glycemic control, and quality of life [4]. These conditions are not merely psychological sequelae; they are increasingly understood to be pathophysiologically intertwined with the metabolic dysregulation of diabetes. Peripheral neuropathy, vascular insufficiency, and systemic inflammation contribute to pain, while prolonged hyperglycemia promotes oxidative stress and advanced glycation end-products that drive nerve injury and ischemic pain. Concurrently, chronic low-grade inflammation characteristic of diabetes is thought to amplify both somatic pain and depressive symptoms [5].

Against this background, ketamine, a dissociative anesthetic with a distinctive pharmacologic profile, has emerged as a candidate for repurposing in multimodal diabetes care. Ketamine’s primary mechanism involves noncompetitive antagonism of N-methyl-D-aspartate (NMDA) receptors, dampening excitatory glutamatergic transmission central to pain processing and the neuroplastic changes associated with depression [6,7]. It secondarily enhances α-amino-3-hydroxy-5-methyl-4-isoxazolepropionic acid (AMPA) throughput and engages downstream signaling, including brain-derived neurotrophic factor (BDNF) and mammalian target of rapamycin (mTOR) pathways, contributing to rapid-acting antidepressant effects. Unlike opioid analgesics, ketamine exerts its analgesic activity primarily through non-opioid mechanisms. Its noncompetitive antagonism on NMDA receptors disrupts central sensitization, reduces spinal “wind-up,” and attenuates hyperalgesia, mechanisms highly relevant to neuropathic and ischemic pain in diabetes. Additional actions, including facilitation of AMPA signaling, inhibition of hyperpolarization-activated cyclic nucleotide-gated (HCN) channels, and modulation of monoaminergic and inflammatory pathways, further differentiate ketamine from opioids and contribute to its opioid-sparing properties.

Pharmacokinetically, ketamine is rapidly absorbed and widely distributed because of its high lipid solubility and a large volume of distribution. It undergoes extensive hepatic metabolism via cytochrome P450 enzymes, yielding active metabolites such as norketamine and hydroxynorketamine (HNK) with distinct receptor affinities and half-lives [8]. These metabolites are increasingly investigated for sustaining antidepressant and anti-inflammatory effects with fewer dissociative adverse events (AEs). Clinical interest has expanded beyond psychiatry and anesthesiology: in 2019 the FDA approved intranasal (IN) esketamine, the S-enantiomer of ketamine, for treatment-resistant depression (TRD) and subsequently for major depressive disorder with acute suicidality [9]. Evidence from preclinical and clinical studies also supports benefits in acute and chronic pain, including neuropathic and ischemic syndromes prevalent in diabetes [10,11]. Notably, ketamine’s analgesic activity is not opioid-receptor dependent, making it attractive for opioid-sparing regimens.

Emerging work suggests ketamine may modulate glucose metabolism and insulin sensitivity, potentially via anti-inflammatory effects or central regulation of energy homeostasis [12]. A recent scoping review of stress-model animals and human trials reported no consistent, sustained alterations in glucose-insulin homeostasis [13]. This is clinically relevant given that several antidepressant and analgesic classes are associated with adverse metabolic profiles. Given the high prevalence of comorbid pain and depression among people with diabetes and their complex pharmacologic needs, ketamine represents a promising, yet still exploratory, adjunct.

This scoping review synthesizes evidence on ketamine/esketamine receptor pharmacology, metabolism, and clinical utility in the context of diabetes and diabetic neuropathy (DN), with attention to metabolic safety. Anticipating model-informed precision dosing, we also delineate a population pharmacokinetic (popPK) and pharmacokinetic-pharmacodynamic (PK-PD) research agenda, highlighting candidate covariates (e.g., eGFR, hepatic function, inflammatory status, HbA1c, genotype, body weight, and co-medications), drug–disease–drug interactions (notably with metformin, GLP-1 receptor agonists, and CYP2B6/CYP3A4 modulators), and pragmatic sparse-sampling windows to quantify variability and inform individualized dosing in diabetic populations. Previous reviews of ketamine have largely examined its antidepressant or analgesic effects in heterogeneous populations, or have discussed metabolic safety without specific attention to diabetes status. In contrast, the present scoping review focuses on ketamine and esketamine use in individuals with diabetes and diabetic animal models, integrating receptor pharmacology, metabolic pathways, and clinical outcomes with a forward-looking diabetes-specific popPK/PK-PD framework. This diabetes-focused synthesis aims to inform both mechanistic hypotheses and pragmatic dosing strategies for this high-risk population.

## 2. Materials and Methods

### 2.1. Protocol and Eligibility

The project was initially registered in PROSPERO as a systematic review (CRD420251015451). During the screening phase, we determined that the available evidence consisted of heterogeneous human and animal studies that were not suitable for quantitative synthesis or for a traditional risk-of-bias appraisal. To more accurately reflect the nature and breadth of the literature, we refined the protocol and conducted review using a scoping approach.

**Figure 1:**
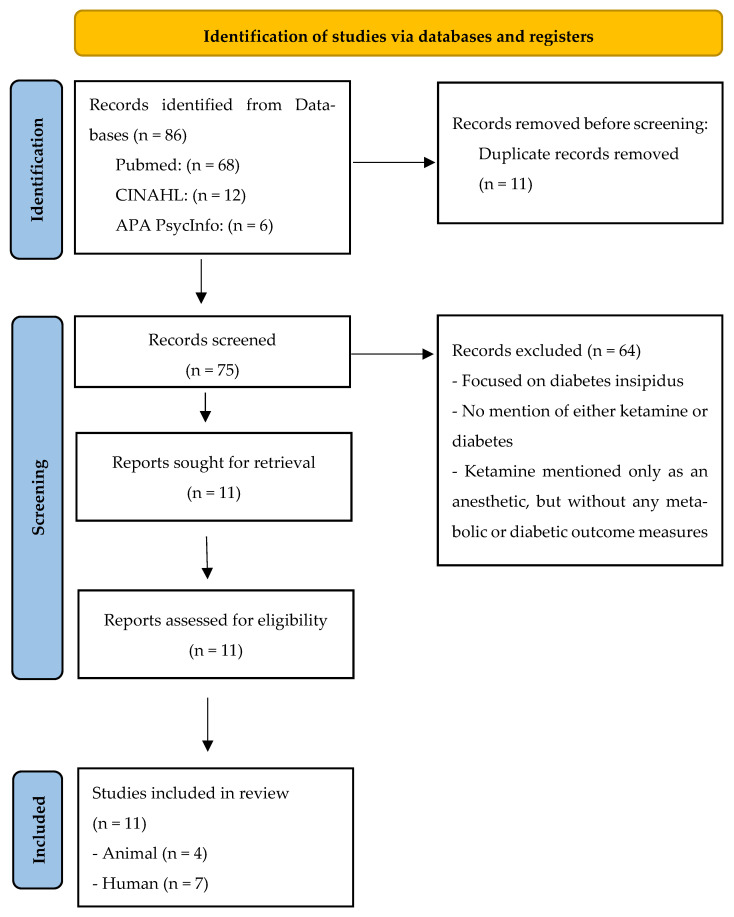
PRISMA-ScR flow diagram of study selection [15]. A total of 86 records were identified (PubMed = 68, CINAHL = 12, APA PsycInfo = 6). After removal of 11 duplicates, 75 records were screened, 11 full texts were assessed for eligibility, and 11 studies were included in the final scoping review. Common exclusion reasons included non-diabetic populations, lack of ketamine exposure, and absence of relevant metabolic or psychiatric outcomes.

This review followed the PRISMA-ScR [14] reporting guideline and uses the updated PRISMA 2020 flow diagram format [15] to illustrate the study selection process (Figure 1). We included human and animal studies that: (a) involved diabetes (type 1 [T1DM], type 2 [T2DM], gestational [GDM], or DN); (b) evaluated ketamine, esketamine, or norketamine by any route or dose; and (c) reported outcomes relevant to depression, pain, inflammation, or glycemic/metabolic regulation. We excluded non-peer-reviewed items, records without accessible full text, studies focused exclusively on recreational misuse without clinical or translational relevance, and studies that did not stratify by diabetes or report metabolically relevant outcomes.

### 2.2. Information Sources and Search Strategy

We searched MEDLINE/PubMed, CINAHL, and APA PsycInfo for records from 1 January 2020 to 31 May 2025 (selected to capture the esketamine era and recent metabolic/safety data). Search strings combined controlled vocabulary and keywords: (“ketamine” OR “esketamine” OR “norketamine”) AND (“diabetes” OR “type 1 diabetes” OR “type 2 diabetes” OR “gestational diabetes” OR “diabetic neuropathy”) AND (“pain” OR “depression” OR “inflammation” OR “glucose metabolism”). Searches were limited to English. We also screened reference lists of included articles. We limited the primary database search to 1 January 2020–31 May 2025, to focus on the esketamine era and contemporary safety data most relevant to current clinical practice. Foundational mechanistic and pharmacologic studies published before 2020 (e.g., early NMDA, catecholaminergic, and metabolic interaction work) were nevertheless identified through backward citation searching and targeted, non-systematic queries and are incorporated narratively in the pharmacology sections.

### 2.3. Study Selection

Records were de-duplicated and screened by two independent reviewers at the title/abstract stage and then at full text using a standardized form in Excel; disagreements were resolved by consensus with a third reviewer. Consistent with a scoping design, we did not calculate inter-rater agreement (e.g., kappa). Yield: 86 records identified; 11 duplicates removed; 75 records screened; 11 full texts assessed for eligibility; 11 studies included. Common exclusion reasons were: non-diabetic population, no ketamine exposure, no relevant outcomes, or non-peer-reviewed publication type.

### 2.4. Data Charting and Synthesis

We charted author/year, study design, population or animal model, diabetes type, ketamine regimen (enantiomer, route, dose), comparators, outcomes (clinical and metabolic), and AEs. Because of heterogeneity in designs and outcomes, we conducted narrative/descriptive synthesis without quantitative meta-analysis or formal risk-of-bias appraisal. Although formal risk-of-bias tools were not applied, consistent with PRISMA-ScR guidance, we conducted a qualitative appraisal of each study’s design features, including sample size, presence of comparator arm, clarity of ketamine exposure, and robustness of outcome measurement. These features are summarized in Table 1 and referenced in the Results to provide context for interpreting the strength and transferability of the available evidence.

## 3. Results

Study set (n = 11). The corpus comprised four human studies (three T1DM case reports/series; one T2DM case report describing ketamine-assisted psychotherapy [KAP]), one randomized controlled trial (RCT) in GDM evaluating post-cesarean analgesia, two narrative reviews on topical ketamine for DN, and four preclinical rodent studies using streptozotocin or diet-induced diabetes models. Table 1 summarizes all included evidence, distinguishing primary human and animal studies from secondary reviews. The table details study design, diabetes context, population or model, clinical indication, ketamine regimen, comparator, outcomes, AEs, and principal findings.

### 3.1. T1DM

Case reports describe rapid antidepressant benefits with intermittent intravenous (IV) ketamine in TRD and note recurrent hypoglycemia during some infusion sessions, highlighting the need for close glucose monitoring in T1DM [16]. Pediatric/anesthesia cases highlight multimodal pain strategies incorporating ketamine while recognizing the broader risk milieu in adolescents with T1DM (e.g., osteopenia, complex regional pain syndrome) [17,18]. Lortrakul and Pattanaseri [16] described a 36-year-old male with T1DM who developed recurrent hypoglycemia during IV ketamine infusions (0.5 mg/kg over 40 min) for TRD. Despite psychiatric improvement, the patient experienced multiple hypoglycemic episodes. The authors emphasized the need for rigorous glucose monitoring in T1DM patients receiving ketamine, due to its potential metabolic impact. Alzahid et al. [18] reported procedural sedation with ketamine in a 17-year-old male with T1DM following orthopedic trauma. Used alongside morphine for closed reduction in an ankle injury, ketamine provided effective analgesia. The case highlighted the elevated risk for complications such as osteopenia and chronic pain in adolescents with T1DM, emphasizing the need for multimodal pain strategies that incorporate careful use of ketamine. Easterly and Taylor [17] presented a case of an adolescent with T1DM, suicidal ideation, and disordered eating. After poor response to conventional anesthesia, the patient was treated with a ketamine-propofol combination, resulting in marked improvement in depressive symptoms. Although anecdotal, this case emphasizes ketamine’s potential rapid antidepressant effects in complex pediatric metabolic-psychiatric profiles.

### 3.2. T2DM

A KAP case in a patient with T2DM and neuropsychiatric comorbidity reported improvements in anxiety, depression, suicidality, and behavioral regulation after a series of IV ketamine infusions and boosters, with no reported AEs; single-case data limit inference on metabolic safety [19]. Harris et al. [19] reported on a 29-year-old male with T2DM, bipolar disorder, and autism spectrum disorder who underwent six IV ketamine infusions followed by two booster sessions. Improvements were observed in affective instability and behavioral regulation. Single-case data limit inference on metabolic safety.

### 3.3. GDM

In a double-blind RCT of women with GDM undergoing cesarean delivery, low-dose esketamine administered via patient-controlled analgesia (with sufentanil) reduced opioid consumption and movement-evoked pain without excess adverse effects, indicating opioid-sparing potential in obstetric care [20].

### 3.4. DN

Two secondary reviews summarize topical ketamine (often combined with amitriptyline) for refractory DN, reporting mixed to modest benefit with fewer systemic adverse effects compared with oral agents; heterogeneity in formulations and small samples limit certainty [21,22]. Rastogi and Jude [21] reviewed adjunctive topical therapies, including 5% ketamine cream, amitriptyline, and clonidine for refractory DN. They noted modest benefits and highlighted the potential for reduced systemic side effects compared to oral agents. Elbeddini et al. [22] compared topical versus oral analgesics and found favorable results for ketamine-containing topicals, particularly in combination with amitriptyline. While findings are preliminary, topical ketamine may offer a safer alternative in patients with systemic comorbidities or poor oral medication tolerance.

### 3.5. Animal Models

Rodent studies suggest context-dependent interactions between ketamine and the diabetic milieu: altered anesthesia dynamics and glucose kinetics [23]; attenuation of ketamine-related hyperactivity, cognitive deficits, and neuroinflammation by GLP-1 receptor agonist co-treatment [24]; worsened neurological outcomes with ketamine–xylazine in ischemic T2DM models mitigated by antidiabetic therapy [25]; and cardiometabolic benefit when ketamine was co-administered with insulin in myocardial ischemia/reperfusion models, including reductions in glucose, hepatic and cardiac enzymes (AST, LDH, CK-MB), and inflammatory markers (IL-1β, IL-6, TNF-α), with improved autophagy regulation [26]. Yilmaz and Dokuyucu [23] studied streptozotocin-induced diabetic rats and found that ketamine anesthesia was associated with faster induction and altered glucose kinetics, suggesting the need for diabetes-specific anesthetic dosing in both preclinical and clinical settings. Sedky et al. [24] evaluated liraglutide, a GLP-1 receptor agonist, in combination with ketamine. In diabetic rats, liraglutide attenuated ketamine-induced hyperactivity, cognitive impairments, and neuroinflammation (e.g., TNF-α elevation), indicating potential synergistic benefits in modulating central inflammatory pathways. Chavda and Patel [25] used a T2DM rat model with induced ischemic stroke (via middle cerebral artery occlusion) and reported worsened neurological outcomes following ketamine-xylazine exposure. However, treatment with anti-diabetic agents (voglibose and saxagliptin) mitigated these effects, highlighting the neuroprotective role of glycemic control during ketamine anesthesia. Tao et al. [26] investigated the cardioprotective effects of ketamine, alone and in combination with insulin, in a diabetic myocardial ischemia/reperfusion injury model. Co-administration led to significant reductions in glucose levels, hepatic and cardiac enzymes (AST, LDH, CK-MB), and inflammatory markers (IL-1β, IL-6, TNF-α), alongside improved autophagy regulation. These findings suggest a cardiometabolic benefit of ketamine when used with insulin in diabetic conditions.

## 4. Discussion

To our knowledge, no prior review has systematically collated ketamine data across T1DM, T2DM, GDM, and DN contexts while simultaneously outlining a diabetes-specific popPK/PK-PD agenda; this is the gap our scoping review seeks to address.

### 4.1. Pharmacology and Population Pharmacokinetics

#### 4.1.1. Pharmacodynamic Profile

**Figure 2:**
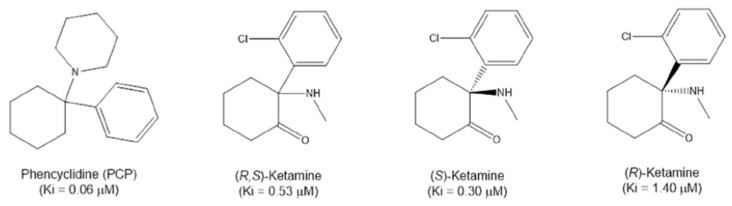
Chemical structures of phencyclidine (PCP), racemic ketamine ((R,S)-ketamine), and its enantiomers ((S)-ketamine and (R)-ketamine), with Ki values indicating NMDA receptor binding affinity. The (S)-enantiomer shows greater potency than the (R)-enantiomer, reflecting clinically relevant differences in pharmacological activity [28].

Ketamine, a chiral derivative of phencyclidine, is administered clinically as a racemic mixture consisting of equal parts (S)-ketamine and (R)-ketamine (Figure 2). These enantiomers display distinct pharmacokinetic and pharmacodynamic properties. The (S)-enantiomer demonstrates a higher affinity for NMDA receptors and is more potent in producing anesthetic and analgesic effects, whereas (R)-ketamine and its downstream metabolites, particularly HNK, are associated with sustained antidepressant and neuroprotective effects with fewer dissociative side effects [27,28]. This enantiomer-specific pharmacology has prompted interest in whether one stereoisomer may offer therapeutic advantages for patients with metabolic comorbidities.

Ketamine’s broad receptor binding profile extends beyond NMDA antagonism. It interacts with opioid, monoaminergic, cholinergic, and purinergic receptors, as well as HCN channels. Such pleiotropic activity may explain the heterogeneity of its clinical effects, particularly in complex populations such as those with diabetes, who present with overlapping psychiatric, neuropathic, and metabolic disturbances.

From a metabolic perspective, ketamine undergoes extensive first-pass hepatic biotransformation. N-demethylation via cytochrome P450 isoenzymes, primarily CYP2B6 and CYP3A4, produces the active metabolite norketamine. Norketamine is further hydroxylated to HNK and dehydroxynorketamine (DHNK), both of which exhibit pharmacological activity distinct from the parent compound. HNK, in particular, has been shown to act through NMDA-independent mechanisms, potentially contributing to antidepressant and anti-inflammatory properties without the psychotomimetic effects of ketamine [27]. This has made HNK a focus of pharmacological interest as a candidate for novel antidepressant development.

#### 4.1.2. Pharmacokinetics with Relevance to Diabetes

Alterations in ketamine pharmacokinetics are increasingly recognized in individuals with diabetes, a population characterized by chronic low-grade inflammation, hepatic insulin resistance, and oxidative stress. These pathophysiological changes can impair hepatic enzyme function, downregulate CYP450 activity (particularly via cytokines such as IL-6 and TNF-α), and potentially reduce metabolic clearance. In parallel, DN may compromise renal elimination of ketamine and its metabolites. Collectively, these factors suggest that patients with diabetes may require dose adjustments or closer therapeutic monitoring, although robust clinical data remain limited.

Preclinical studies have begun to elucidate these alterations. Yilmaz and Dokuyucu [23] reported faster induction times in streptozotocin-induced diabetic rats administered ketamine anesthesia, suggesting impaired absorption or altered distribution kinetics. Tao et al. [26] further demonstrated that ketamine modulated cardiometabolic injury in a diabetic myocardial ischemia/reperfusion model. In this context, ketamine reduced levels of hepatic and cardiac injury markers (AST, LDH, CK-MB) and suppressed inflammatory cytokines (IL-1β, IL-6, TNF-α). These effects were enhanced when ketamine was co-administered with insulin, pointing to potential synergistic benefits in modulating autophagy and systemic inflammation.

Pharmacokinetics vary significantly by route of administration. IV ketamine provides 100% bioavailability with an initial distribution half-life of less than one minute. Intramuscular (IM) injection achieves ~93% bioavailability, with onset of action within five minutes [29]. IN administration has a bioavailability of ~50%, while intrarectal administration yields ~25% [30]. Oral bioavailability is substantially lower, ~20%, due to extensive first-pass metabolism [31]. Epidural and intrathecal routes are associated with systemic absorption, which may explain psychodysleptic effects observed after neuraxial administration [32].

Enantiomer-specific differences further complicate the pharmacokinetics of ketamine. (S)-ketamine is approximately four-fold more potent than (R)-ketamine and, in some contexts, twice as effective as the racemic mixture [33]. Its oral bioavailability ranges between 8 and 11%, lower than the racemate, reflecting its higher susceptibility to first-pass metabolism [34]. When administered alone, (S)-ketamine also demonstrates higher systemic clearance, suggesting possible inhibitory interactions between enantiomers during racemic administration. Both in vitro and in vivo studies confirm that inhibition of CYP3A4, CYP2B6, and CYP2C9 reduces N-demethylation of ketamine, reinforcing the central role of these enzymes in both absorption and elimination [33].

#### 4.1.3. Elimination and Clearance

Ketamine exhibits relatively low plasma protein binding (10–30%) but high systemic clearance (60–147 L/h/70 kg) and a short elimination half-life of 2–4 h. Renal excretion accounts for clearance of both parent compound and metabolites, with only small amounts excreted unchanged: ~2% as ketamine, ~2% as norketamine, and ~16% as DHNK. The majority (~80%) of elimination occurs as glucuronidated conjugates of HNK and DHNK excreted in urine and bile [27].

This elimination profile highlights ketamine’s clinical relevance in populations with metabolic or renal dysfunction. Patients with diabetes, particularly those with DN, may demonstrate altered clearance and metabolite accumulation, increasing the risk of both therapeutic variability and toxicity. The short half-life features ketamine’s rapid onset and offset of action, but variability in metabolism and renal function may significantly impact dosing considerations in diabetic populations.

#### 4.1.4. Diabetes-Specific popPK/PK-PD Framework for Ketamine (Proposed for Future Research)

popPK/PK-PD modeling represents core methodologies in translational pharmacology and aligns closely with the scope of *Pharmaceutics*, which emphasizes quantitative approaches for therapeutic optimization. In individuals with diabetes, physiological alterations, including chronic inflammation, variable hepatic and renal function, and complex polypharmacy, may introduce substantial variability in ketamine disposition and response. Although no popPK/PK-PD analyses were conducted in this scoping review, the following proposed framework outlines how future modeling efforts could characterize these covariate influences, support model-informed precision dosing (MIPD), and inform individualized ketamine therapy in diabetic populations.

We outline a prospective diabetes-specific popPK/PK-PD modeling framework that could (i) quantify inter- and intra-individual variability in parent ketamine and metabolic exposure, (ii) link exposure to antidepressant, analgesic, hemodynamic, and glycemic effects, and (iii) generate simulations to guide dose optimization in clinical practice.

(1) Structural models
  i. Racemic IV ketamine: Future models may use a two-compartment disposition structure with linear elimination. Between-subject variability (BSV) could be modeled as log-normal on clearance (CL), central volume (V1), intercompartmental clearance (Q), and peripheral volume (V2). Inter-occasion variability (IOV) on CL and V1 may be necessary for repeated infusions. If chiral bioanalysis is available, stereospecific extensions (separate CL and V1 for S- and R-ketamine and formation clearances) are recommended; otherwise, the racemate may be modeled as a single analyte.
  ii. IN esketamine: A prospective model could include an absorption compartment with absolute bioavailability (F), first-order absorption rate (Ka), and a lag time to account for device- and administration-related variability. Distribution would follow a two-compartment model. Zero-order input or mixed-order input may be evaluated in sensitivity analyses for mucosal saturation or device pooling.
  iii. Metabolites (norketamine, HNK): Joint parent–metabolite modeling using metabolic formation clearances and first-order elimination could be used. If chiral measurements are available, parallel S- and R-norketamine/HNK pathways are recommended. This structure would allow unbiased estimation of parent CL and enable exposure–response assessment for metabolites with putative antidepressant and anti-inflammatory activity.
  iv. Error models and below-limit-of-quantification (BLQ): Combined additive–proportional residual error models may be appropriate. BLQ data could be handled using the M3 likelihood method.
  v. Allometry: Theory-based allometric scaling (CL and Q scaled to body weight^0.75; V1, V2 to weight^1.0) may be applied, with sensitivity analyses for extreme BMI values.
(2) Pre-specified covariates

Future diabetes-focused popPK/PK-PD studies should predefine covariates that reflect metabolic disease heterogeneity and common co-therapies. Continuous covariates may be included using centered power functions; categorical covariates through fractional shifts on the log-scale.

  i. Size: Body weight (kg) via allometry; BMI may be an exploratory predictor of F and Ka for IN dosing.
  ii. Renal function: eGFR may influence metabolite clearances and, secondarily, parent CL. A nephropathy indicator (yes/no) may capture non-linear impairment beyond eGFR.
  iii. Hepatic function: ALT, AST, and bilirubin could inform parent CL and F (IN). Hepatic impairment categories (mild/moderate) may also be incorporated.
  iv. Inflammation: CRP and IL-6 may serve as time-varying covariates on CL to represent cytokine-mediated CYP down-regulation (e.g., CL = CL_pop × (1 − θ_IL6 × (IL-6 − IL-6_ref)).
  v. Glycemic control: HbA1c, diabetes type/duration, and insulin regimen may influence CL/F and PD baselines.
  vi. Pharmacogenomics: Variants such as CYP2B6*6, CYP3A4*22, BDNF Val66Met, and COMT Val158Met may be evaluated as modifiers of popPK/PK-PD responses.
  vii. Concomitant medications: Strong CYP3A4/2B6 inhibitors/inducers and antidiabetic therapies (metformin, GLP-1 receptor agonists, SGLT2 inhibitors, insulin) should be examined as covariates.
  viii. Route/formulation: Indicators for IV/IN/PO/IM may be included; for IN, device type and administration technique (unilateral vs. bilateral) could be modeled.

Shrinkage diagnostics would be required to ensure stable covariate interpretation, and non-linearities may be examined with covariate-ETA plots and generalized additive models.

(3) Clinic-feasible sampling design

Where feasible, co-sampling of glucose, blood pressure (BP)/heart rate (HR), and discussion scales at matched time points could enable PK–glycemia and PK–hemodynamic modeling.

  i. IV TRD infusions: Pre-dose, 10–15 min, end infusion (~40–50 min), and 2–4 h.
  ii. IN esketamine: ~15, 45, 120, and 240 min.

(4) PK–PD endpoints and models

Proposed endpoints include:

  i. Antidepressant response: MADRS/HDRS with E_max or indirect response models.
  ii. Analgesia: Pain VAS or neuropathy scores using E_max or ordered categorical models.
  iii. Hemodynamics: BP/HR linked to concentrations with effect compartments.
  iv. Glycemia: Indirect response models for glucose turnover with optional mixture components.
  v. Safety: Dissociation, urinary symptoms, cognitive change, LFTs, and eGFR as PD outcomes.

(5) Estimation, qualification, and missing data

Future models could be estimated in NONMEM, Monolix, or Stan using FOCEI or SAEM. Model qualification may include goodness-of-fit, VPC/pcVPC, bootstrapping, and internal validation. BLQ data could be handled via M3; missing covariates via prespecified imputation rules.

(6) Simulation and decision support

Monte Carlo simulations may quantify:

  i. Drug–drug interactions (DDIs) (strong CYP inhibitors/inducers, grapefruit juice).
  ii. Disease-state scenarios (eGFR strata, CRP/IL-6 quartiles, HbA1c levels).
  iii. MIPD nomograms for safe dosing across patient subgroups.
  iv. Benefit–risk curves linking exposure to clinical response and AEs.

(7) Operational considerations

Future studies should prioritize standardized bioanalytics (including chiral assays), precise time-stamping for PK-PD alignment, and feasible sparse sampling schedules tailored to diabetic patients.

### 4.2. Systemic Effects

Short-term, low-dose IV ketamine for TRD is generally well tolerated, with transient BP/HR elevations that resolve within hours [35]. Chronic/high-dose exposure and misuse raise risks across organ systems: cardiac dysfunction [36], urologic toxicity including ulcerative cystitis and obstructive uropathy [37,38], and neurocognitive/dissociative effects [39,40]. Given the high prevalence of cardiovascular disease (CVD), chronic kidney disease (CKD), and cognitive vulnerability in diabetes, risk may be amplified (supporting structured monitoring).

#### 4.2.1. Cardiovascular Effects

In a prospective observational study, Szarmach et al. [35] examined eight IV ketamine infusions (0.5 mg/kg over 40 min) in patients with TRD and stable somatic comorbidities. The treatment produced transient elevations in HR and BP, which resolved within hours. These hemodynamic responses are clinically important since CVD is a major comorbidity in T2DM, affecting 32.2% of individuals globally. Chronic exposure or high doses of ketamine appear more concerning. Saliba [36] reported ketamine-induced acute systolic heart failure, potentially related to direct myocardial depressant effects. Historical case reports have documented stress cardiomyopathy following perioperative ketamine use [41]. In a related observational study, Szarmach et al. [42] noted that repeated low-dose IV ketamine infusions, when administered adjunctively with psychotropic medications, were associated with overall reductions in systolic blood pressure (SBP) and respiratory rate over time. While reassuring, these findings also highlighted the need for caution in patients with comorbid hypertension or autonomic dysfunction, conditions commonly seen in diabetes.

#### 4.2.2. Renal Effects

Chronic or high-dose ketamine exposure has been linked to a spectrum of urological complications, most notably ketamine-induced ulcerative cystitis. This condition is characterized by urinary urgency, dysuria, and hematuria, with potential progression to obstructive uropathy and hydronephrosis. Repeated episodes of vesicoureteral reflux resulting from bladder wall instability may cause bilateral renal injury. Tsai et al. [37] reported that patients with advanced bladder pathology from chronic ketamine exposure were five times more likely to develop progressive renal dysfunction, and a proportion required hemodialysis for end-stage renal disease. More recently, Brucculeri et al. [38] described the first reported case of acute interstitial nephritis requiring renal replacement therapy following repeated IV ketamine infusions. Although the true prevalence is uncertain due to the absence of routine renal biopsies, these findings underscore the nephrotoxic potential of ketamine, particularly in patients with preexisting renal impairment such as diabetic nephropathy.

#### 4.2.3. Neurocognitive Effects

Ketamine’s dissociative and psychotomimetic properties, while typically transient at therapeutic doses, remain a concern for patients with diabetes, who may already be vulnerable to cognitive decline secondary to microvascular complications and chronic hyperglycemia. Cognitive dysfunction in this population could be exacerbated by ketamine-induced perceptual disturbances, memory impairment, or depersonalization, emphasizing the importance of comprehensive neurocognitive monitoring during treatment.

#### 4.2.4. Overall Systemic Profile

Taken together, ketamine’s systemic effects span multiple organ systems. While subanesthetic doses in controlled clinical settings appear relatively safe, chronic use, higher dosing, or misuse pose substantial risks. For individuals with diabetes, these risks may be amplified by the high prevalence of cardiovascular, renal, and neurocognitive comorbidities. The dual nature of ketamine, as both a promising therapeutic and a drug of abuse, highlights the need for pharmacovigilance, long-term safety studies, and patient-specific risk stratification in clinical practice.

### 4.3. Clinical Applications in Diabetes

#### 4.3.1. Neuropathic Pain

Ketamine can interrupt central sensitization and hyperalgesia via NMDA antagonism. Infusion-based protocols show short-term analgesic efficacy in refractory neuropathic pain, though dosage/duration heterogeneity limits standardization [43,44,45]. DN-focused evidence for topical ketamine is mixed-to-modest with better systemic tolerability; consider as an adjunct in refractory DN [21,22].

Neuropathic pain, one of the most common and debilitating complications of diabetes, arises from injury or dysfunction of the peripheral or central nervous system. DN can manifest as peripheral neuropathy with distal symmetric pain and sensory loss, autonomic neuropathy affecting cardiovascular reflexes and bladder control, proximal neuropathy producing severe hip and thigh pain, or focal neuropathy involving cranial or peripheral nerves. These conditions result from prolonged hyperglycemia, oxidative stress, and microvascular injury. Conventional analgesics for DN, including pregabalin, duloxetine, and tricyclic antidepressants, often provide incomplete relief and are limited by side effects. Ketamine’s unique pharmacology, primarily its non-competitive antagonism of the NMDA receptor, allows it to interrupt central sensitization, prevent hyperalgesia, and modulate descending excitatory pathways implicated in neuropathic pain.

A systematic review by McMullin et al. [43] assessed recent advances in infusion-based therapies for neuropathic pain and identified ketamine as a promising option. However, variability across clinical trials in dosage, infusion duration, and protocols made comparisons difficult. Still, the review concluded that IV ketamine provided short-term analgesic efficacy and recommended individualized titration protocols with pre-infusion screening to improve safety and cost-effectiveness. Evidence specific to DN is limited but encouraging. Rastogi and Jude [21] reported that adjunctive topical ketamine formulations, often in combination with amitriptyline or clonidine, provided modest benefit in refractory DN with reduced systemic side effects. Elbedinni et al. [22] compared topical and oral analgesics, concluding that ketamine-containing creams may be favorable in patients with comorbidities that limit oral therapy. A meta-analysis by Pereira et al. [44] found significant reductions in neuropathic pain intensity following ketamine infusion compared with standard care, including reductions sustained at one week and one month (*p* < 0.00001). Further support comes from Bosma et al. [45], who evaluated daily IV ketamine infusions over five consecutive days in refractory neuropathic pain. At follow-up, approximately half of participants reported at least a 30% reduction in pain intensity. Although DN-specific cohorts were limited, these results suggest potential translatable benefits. Smaller case studies also highlight ketamine’s analgesic role in DN, especially for patients with severe or treatment-resistant symptoms.

Despite these promising findings, gaps remain. Longitudinal data are lacking, and the chronicity of DN raises questions about repeated dosing, cumulative toxicity, and long-term safety. Diabetic patients may be at greater risk of hepatic and renal impairment, autonomic dysfunction, and systemic inflammation—all of which may influence ketamine metabolism and adverse event profiles. Neurotoxicity, cardiovascular risk, and urological complications also warrant caution. At present, ketamine should be reserved for refractory DN in carefully selected patients, ideally managed within multidisciplinary pain programs or controlled clinical protocols.

#### 4.3.2. Depression in Diabetes

The diabetes–depression relationship is bidirectional and biologically plausible (HPA activation, inflammation, cerebrovascular changes); depression undermines self-management and adherence [46,47,48,49]. Ketamine’s rapid antidepressant effects (hours to days) may provide stabilization in TRD and re-engage patients in diabetes care [9,50,51,52]. However, diabetes may modify exposure/response, and long-term safety in diabetic cohorts remains under-characterized, highlighting the need for popPK/PK-PD anchored trials.

The interplay between depression and diabetes is multifactorial, involving both psychosocial stressors and shared biological mechanisms. The burden of chronic disease, stigma, and reduced healthcare access contributes to psychological distress. In addition, dysregulation of the hypothalamic–pituitary–adrenal (HPA) axis, chronic low-grade inflammation, oxidative stress, and cerebrovascular dysfunction have been implicated in the pathogenesis of both diabetes and depression [48,53]. Hyperglycemia itself has neuropsychiatric implications. MRI-based studies demonstrate elevated levels of prefrontal glutamate–glutamine–GABA in individuals with T1DM, correlating with depressive symptoms [47]. This glutamatergic dysregulation parallels findings in MDD, supporting the rationale for NMDA-targeted interventions such as ketamine. Conversely, untreated depression negatively affects diabetes self-management, including adherence to medications, glucose monitoring, dietary choices, and clinic attendance. A meta-analysis of 47 studies confirmed that depression is significantly associated with treatment nonadherence in diabetes [47]. This reciprocal relationship exacerbates glycemic variability and increases the risk of complications such as nephropathy, neuropathy, and CVD [46,49].

Depression is also a risk factor for incident T2DM, independent of demographic or lifestyle factors. Proposed mechanisms include HPA axis activation, hypercortisolemia, insulin resistance, and altered adipokine signaling [54,55]. In recognition of this bidirectional relationship, the American Diabetes Association [56] now recommends routine depression screening in patients with diabetes, particularly those with poor glycemic control or complications. Traditional antidepressants, such as SSRIs, SNRIs, and TCAs, are limited by delayed onset (several weeks) and incomplete efficacy. Approximately 20–30% of MDD patients meet criteria for TRD. The limitations of monoaminergic therapies have intensified interest in ketamine as a rapid-acting antidepressant. Unlike conventional agents, ketamine induces symptom relief within hours through NMDA receptor antagonism, AMPA receptor activation, mTOR signaling, and BDNF-mediated synaptic plasticity. Singh et al. [57] reported that a single IV infusion of ketamine (0.5 mg/kg over 40 min) can produce significant improvements lasting up to seven days. Repeated dosing can prolong therapeutic benefit, although optimal maintenance regimens remain undefined.

For diabetic patients with TRD, ketamine’s rapid effects may serve as both an emergency intervention (e.g., suicidal ideation) and a means of re-engaging patients in chronic disease management. By alleviating depressive symptoms, ketamine could indirectly improve adherence, self-care, and glycemic control, potentially reducing hospitalizations and complications. Nevertheless, important uncertainties remain. Diabetes alters ketamine pharmacokinetics and may modify antidepressant efficacy and safety. Poor glycemic control may affect CNS availability, while hepatic inflammation or renal dysfunction could alter clearance of ketamine and its metabolites. Long-term risks (including neurotoxicity, urological toxicity, cardiovascular instability, and potential for misuse) are not yet adequately characterized in diabetic populations. Consequently, ketamine should only be considered in carefully selected individuals with TRD and diabetes, administered under specialist supervision with comprehensive metabolic, cardiovascular, and psychiatric monitoring.

### 4.4. Metabolic Implications and Therapeutic Limitations

Evidence on ketamine’s glycemic effects is variable across settings: both hyper- and hypoglycemia have been observed (animal anesthesia, perioperative contexts, and TRD infusions in T1DM case reports). Mechanisms may include adrenergic signaling, hepatic blood-flow/CYP modulation, and insulin/gluconeogenesis dynamics; in some models, insulin co-administration attenuates cardiac I/R injury and inflammatory markers [26]. Because ketamine undergoes extensive hepatic metabolism and is frequently administered alongside glucose-lowering therapies, clinically relevant pharmacokinetic and pharmacodynamic interactions may substantially influence both antidepressant efficacy and metabolic stability in patients with diabetes (Table 2).

Preclinical findings suggest metformin may blunt sustained antidepressant-like effects [58], warranting clinical evaluation given its first-line role. Early cardiomyocyte data in insulin resistance point to calcium handling/oxidative stress pathways [12]. Neurocognitive risks (transient dissociation, memory effects) must be weighed in patients already at risk for cognitive decline [40,61,62].

Ketamine’s pharmacological activity is influenced by metabolic status and its interactions with antidiabetic therapies, making its use particularly complex in individuals with diabetes. Chuang et al. [58], for example, demonstrated in a rodent model that metformin attenuated ketamine’s sustained antidepressant effects, raising the possibility of a pharmacodynamic interaction between two commonly used agents. Because metformin is the first-line therapy for T2DM, these findings underscore the need for caution when considering ketamine co-prescription, as antidepressant efficacy may be compromised. Similar concerns extend to other glucose-lowering medications, including insulin sensitizers and GLP-1 receptor agonists, which may alter ketamine’s pharmacokinetic and pharmacodynamic profile in ways that remain poorly understood. Clinical observations further reinforce these concerns. Lortrakul et al. [16] described recurrent hypoglycemia in a 36-year-old male with T1DM undergoing IV ketamine infusions for treatment-resistant depression, despite marked psychiatric improvement. This contrasts with reports of transient hyperglycemia following low-dose ketamine, suggesting a biphasic, dose-dependent effect on glucose regulation. The mechanisms underlying these divergent outcomes appear multifactorial, involving adrenergic signaling, hepatic metabolism, and renal-hormonal pathways. Hyperglycemia has been attributed to α2-adrenergic receptor activation, whereas β-adrenergic receptor activity may mediate hypoglycemia. Ketamine may also alter hepatic blood flow and cytochrome P450 activity, thereby influencing insulin clearance, gluconeogenesis, and systemic glucose regulation [12,27]. Case reports of ketamine-induced diabetes insipidus [63] suggest that disruptions in vasopressin-mediated fluid balance may further destabilize metabolic and electrolyte control in susceptible individuals.

Beyond direct effects on glucose, ketamine may also impact broader cardiometabolic processes. Ramirez et al. [12] demonstrated that ketamine exposure in insulin-resistant cardiomyocytes disrupted intracellular calcium signaling and increased mitochondrial oxidative stress, mechanisms that could predispose to adverse cardiovascular outcomes. These findings are particularly concerning in T2DM, where insulin resistance and low-grade inflammation already heighten cardiovascular risk. At the same time, ketamine’s known suppression of pro-inflammatory cytokines such as IL-6 and TNF-α could provide dual benefits by alleviating depressive symptoms and reducing systemic inflammation. Yet in diabetic populations, this immunomodulatory action might interfere with cytokine-mediated insulin signaling and glucose uptake, with effects that likely vary depending on disease stage, comorbidities, and concurrent medications. Neurocognitive consequences add another layer of complexity. Ketamine’s dissociative and psychedelic effects (such as perceptual distortion, depersonalization, and short-term memory impairment) are well documented [17,39] and pose particular risks to individuals with diabetes, who are already more vulnerable to cognitive impairment from chronic hyperglycemia and microvascular damage [40,61]. These risks may compound, raising concerns about additive neurocognitive burden. Nevertheless, preliminary findings also suggest potential protective effects. Ketamine’s ability to modulate glutamatergic neurotransmission and inflammatory signaling may mitigate neuronal damage and even improve insulin sensitivity in certain contexts [12,62], although such effects appear highly context-dependent, varying with dose, route, and comorbid metabolic dysfunction.

**Figure 3:**
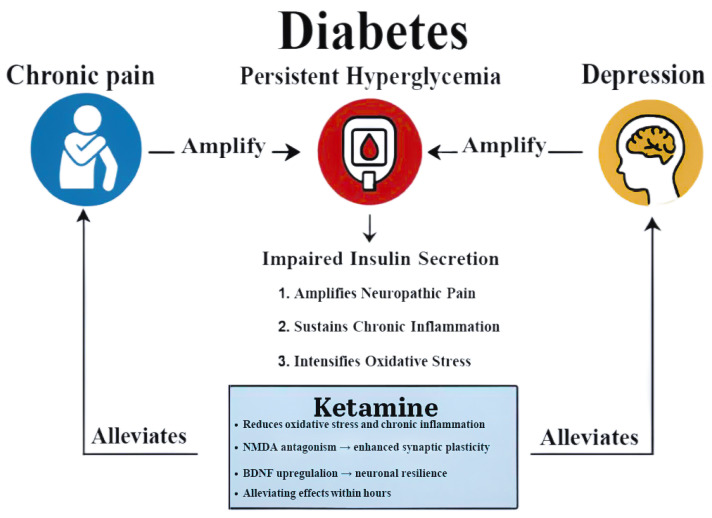
Conceptual framework linking diabetes, chronic pain, and depression with ketamine’s therapeutic potential. Persistent hyperglycemia and impaired insulin signaling amplify neuropathic pain and depressive symptoms through shared inflammatory and neuroendocrine pathways. Ketamine may attenuate these processes by modulating glutamatergic transmission, suppressing pro-inflammatory cytokine activity, and enhancing neuroplasticity via NMDA receptor antagonism and downstream BDNF–mTOR signaling, thereby disrupting the bidirectional amplification between metabolic dysregulation, pain, and depression. This figure was created by the authors for this study.

The current body of evidence indicates that ketamine exerts complex and sometimes unpredictable effects on metabolic regulation in individuals with diabetes. Its rapid antidepressant and analgesic benefits remain promising, but these must be weighed against risks of glycemic volatility, pharmacologic interactions, cardiovascular stress, and cognitive side effects. Figure 3 illustrates the interconnected pathways linking diabetes, chronic pain, and depression, and the potential modulatory role of ketamine. Persistent hyperglycemia contributes to impaired insulin signaling, oxidative stress, and systemic inflammation, which in turn amplify both neuropathic pain and depressive symptoms. These conditions exacerbate one another through overlapping neuroendocrine and inflammatory mechanisms, worsening metabolic control. Ketamine may alleviate this triad by modulating glutamatergic transmission, suppressing pro-inflammatory cytokine activity, and enhancing neuroplasticity via NMDA receptor antagonism and downstream BDNF-mTOR signaling. Through these mechanisms, ketamine could attenuate the bidirectional amplification between pain, depression, and diabetes-related metabolic dysfunction.

While preclinical models provide mechanistic insights into ketamine’s analgesic, antidepressant, and metabolic effects, they cannot fully replicate the complexity of human disease. Translation to clinical practice requires careful interpretation, particularly given the heterogeneity of patient populations and the multifactorial pathophysiology of diabetes. Future research should therefore prioritize pharmacokinetic and pharmacodynamic modeling in diabetic populations, with particular attention to hepatic, renal, and inflammatory variables; clinical trials designed to monitor glucose homeostasis and insulin sensitivity during treatment; careful evaluation of interactions with first-line antidiabetic agents such as metformin, GLP-1 receptor agonists, and SGLT2 inhibitors; and long-term studies that assess whether ketamine has disease-modifying effects in DN or contributes to detrimental metabolic consequences. Key priorities for future RCTs of ketamine in diabetes include assessment of (a) glycemic variability and long-term metabolic stability, (b) cognitive outcomes relative to baseline neurovascular integrity, (c) pharmacologic interactions with antidiabetic and antihypertensive agents, and (d) renal function given diabetes-related vulnerability and ketamine’s clearance pathway. Collectively, these domains are critical for establishing safe, effective and individualized ketamine use in diabetes care.

#### Mechanistic Considerations for Hyperglycemic and Hypoglycemic Responses

Reports of both transient hyperglycemia and recurrent hypoglycemia following ketamine administration likely reflect dose-, context-, and disease-dependent interactions among autonomic tone, hepatic glucose output, and insulin dynamics, mediated primarily through adrenergic and, to a lesser extent, opioid pathways. At lower anesthetic or subanesthetic doses, activation of the sympathetic nervous system and counter-regulatory hormonal responses tends to predominate, resulting in increased hepatic glucose production and transient hyperglycemia. In contrast, hypoglycemia may emerge when specific adrenergic pathways are blocked, blunted, or dysregulated, unmasking opposing effects on insulin secretion, insulin clearance, and peripheral glucose utilization, particularly in susceptible individuals with diabetes.

Experimental evidence indicates that ketamine stimulates central sympathetic outflow, facilitating the release of norepinephrine and epinephrine. These catecholamines elevate blood glucose by promoting hepatic glycogenolysis and gluconeogenesis, suppressing insulin secretion, and enhancing lipolysis [64]. In animal models, low-dose ketamine–associated hyperglycemia has been shown to involve α_2_-adrenoceptor–mediated mechanisms [65]. Increased hepatic glucose production appears to be the primary pathway translating autonomic activation into hyperglycemia. The sympathetic nervous system communicates with the liver through direct hepatic sympathetic innervation, circulating epinephrine acting on hepatic adrenoreceptors, and glucagon-mediated signaling. Consistent with this framework, the magnitude of epinephrine release has been shown to correlate closely with the severity of hyperglycemia [59].

By contrast, direct mechanisms underlying ketamine-associated hypoglycemia are less well defined. During hypoglycemic stress, the autonomic nervous system plays a central role in coordinating neuroendocrine, metabolic, and immunologic responses aimed at restoring euglycemia [66]. One plausible explanation for ketamine-associated hypoglycemia is differential modulation of adrenergic signaling, particularly in contexts where α_2_-adrenoceptor pathways are inhibited or dysregulated. Under such conditions, ketamine’s interactions with opioid receptors and β-adrenergic pathways may shift the balance toward increased insulin action or enhanced peripheral glucose utilization. Additional contributing factors may include changes in hepatic blood flow, altered insulin clearance, and cytokine-mediated effects on insulin signaling and gluconeogenic enzymes.

These mechanisms are likely amplified in patients with diabetes receiving exogenous insulin or intensive glucose-lowering therapy, where background insulin dosing is fixed, and endogenous counter-regulatory capacity may already be impaired. In such settings, higher or repeated ketamine exposures may unmask a tendency toward delayed or recurrent hypoglycemia, providing a mechanistic explanation for the heterogeneous glycemic responses observed across perioperative, psychiatric and preclinical contexts, as well as in clinical case reports involving T1DM and intensive insulin regimens.

### 4.5. Place in Therapy: Model-Informed, Multidisciplinary, and Equitable

As ketamine spreads beyond anesthesia into TRD and chronic pain, integration into diabetes care should be selective and model-informed. While promising, its integration into diabetes care must be framed within the principles of personalized medicine, risk mitigation, and equity. One forward-looking strategy is to incorporate precision medicine approaches that guide individualized ketamine therapy. Precision strategies include pharmacogenomic testing (CYP2B6/CYP3A4; BDNF; COMT) and covariate-guided dosing using popPK/PK-PD. Pharmacogenomic testing may help identify polymorphisms in genes that influence ketamine metabolism, antidepressant efficacy, or susceptibility to neurocognitive side effects [27]. Such insights could inform personalized dosing and monitoring strategies. Equally important is tailoring therapy to the patient’s broader clinical profile (HbA1c levels, vascular complications, depressive symptom burden, and neuropathic pain severity) to ensure that therapeutic benefits are carefully weighed against risks. Future RCTs must include diabetic cohorts, stratify by HbA1c/eGFR/inflammation, capture chiral/metabolite exposures, and co-measure glycemia and safety.

Despite growing interest, the current evidence base remains limited, as many clinical trials of ketamine exclude participants with metabolic disorders. This has left a significant gap in understanding its safety and efficacy in diabetes. Future RCTs should explicitly include diabetic populations and stratify them by metabolic variables such as HbA1c, insulin sensitivity, and inflammatory biomarkers (e.g., IL-6, CRP). These studies should assess the acute and chronic effects of ketamine on glucose homeostasis, evaluate cognitive outcomes relative to baseline neurovascular health, examine drug–drug interactions with commonly prescribed antidiabetic and antihypertensive agents, and determine long-term renal and cardiovascular outcomes given ketamine’s elimination profile and diabetes-related organ vulnerability. Until such data are available, ketamine use in diabetic populations should be restricted to carefully monitored, interdisciplinary settings. Endocrinologists are needed to oversee glycemic control and metabolic risks, psychiatrists to evaluate depressive response and monitor for dissociative or addictive effects, pharmacists to anticipate drug–drug interactions with glucose-lowering therapies, and primary care providers to ensure continuity of care and integration into long-term management. This collaborative model allows for comprehensive risk management across metabolic, psychiatric, cardiovascular, and renal domains.

The expanding use of ketamine also raises important issues of access and equity. Many ketamine clinics operate outside of insurance coverage, creating financial barriers for socioeconomically disadvantaged patients, who are often the same populations disproportionately affected by both diabetes and depression. To address these disparities, future research and policy initiatives must prioritize the inclusion of racially and socioeconomically diverse participants in clinical trials, advocate for insurance reimbursement of evidence-based ketamine protocols, and develop culturally tailored education materials to support informed consent and shared decision-making. Care delivery should be multidisciplinary (endocrinology, psychiatry, pain, pharmacy, primary care) and equity-minded, as access to ketamine is often limited by coverage and cost. Ketamine is unlikely to replace existing therapies; its role is as a bridge/adjunct for refractory depression or DN when conventional options fail, with vigilant monitoring and tapering plans.

Looking ahead, the role of ketamine in diabetes care is unlikely to involve wholesale replacement of existing therapies. Rather, it may serve as a bridge or adjunctive therapy in select patients, particularly those with refractory depression or neuropathic pain who have exhausted conventional options. In such cases, ketamine’s rapid antidepressant and analgesic effects may stabilize patients and improve adherence to chronic disease management, while longer-term strategies address underlying metabolic and psychiatric conditions. The clinical promise of ketamine lies in its ability to target overlapping psychiatric and pain-related complications in diabetes, yet its multifaceted pharmacology, unpredictable metabolic effects, and potential for adverse outcomes demand rigorous investigation and cautious, individualized use. Until more definitive evidence emerges, ketamine’s place in therapy should remain limited to specialized settings under multidisciplinary supervision.

#### Practical Monitoring and Prescribing Considerations in Diabetes

For the limited patients with diabetes in whom ketamine is considered, a cautious, protocolized approach is warranted. In practice, this begins with careful patient selection and baseline risk stratification. Ketamine should be reserved for clearly defined indications, typically TRD or refractory DN, and used only after first-line therapies have been adequately attempted and optimized and, preferably, within a shared plan involving psychiatry, pain management, endocrinology and primary care [21,43,44]. Baseline evaluation should characterize diabetes type and duration, history of DKA or hyperosmolar crises, episodes of severe hypoglycemia or hypoglycemia unawareness, and current insulin and non-insulin regimens, including sulfonylureas, GLP-1 receptor agonists, and SGLT2 inhibitors [1,56]. Cardiovascular and renal risk assessment is essential given the high prevalence of coronary artery disease, heart failure, autonomic neuropathy, and DN in this population, together with ketamine’s known hemodynamic and urologic liabilities [12,35,37,42]. A focused neurocognitive and psychiatric history, including prior psychosis and substance use disorder, is similarly important, given ketamine’s dissociative profile and the elevated background risk of cognitive decline in diabetes [40,62,67].

Metabolic monitoring should explicitly reflect ketamine’s bidirectional glycemic effects, with reports of both transient hyperglycemia and recurrent hypoglycemia in diabetes [16,64]. At a minimum, point-of-care capillary glucose should be assessed pre-dose and at the end of infusion or peak effect during early treatment sessions, with additional measurement 1–2 h post-dose in individuals with T1DM or those receiving intensive insulin therapy, given reports of delayed-onset hypoglycemia [16]. Sessions should be deferred when pre-dose glucose is markedly abnormal until corrected, with thresholds individualized according to local diabetes-management protocols [56]. In patients using SGLT2 inhibitors, with a recent history of DKA, or during intercurrent illness, blood or urine ketones should be evaluated when hyperglycemia or otherwise unexplained symptoms occur to detect DKA, including euglycemic presentations [56]. Close communication with diabetes care teams regarding short-term adjustments of basal and prandial insulin on treatment days may further reduce hypoglycemia risk in high-risk T1DM.

Subanesthetic IV ketamine and IN esketamine are associated with transient increases in BP and HR, typically peaking near the end of infusion or approximately 40 min post-dose and resolving within hours [9,35,51]. Although uncommon, cases of acute systolic heart failure and stress cardiomyopathy have been reported in susceptible individuals [36,41]. In diabetic populations, where CVD, autonomic neuropathy, and silent ischemia are common, pre-session documentation of BP, HR, and heart failure symptoms should be routine, with optimization of background antihypertensive and cardioprotective therapy when feasible. During treatment, vital-signs should be monitored at regular intervals and patients should be observed post-dose until parameters return toward baseline [68,69]. Brief, clinic-feasible dissociation scales can be used during early sessions to document onset, peak, and resolution of dissociative symptoms, helping distinguish expected transient effects from prolonged cognitive or behavioral changes, particularly in older adults or those with pre-existing cognitive impairment [40,52,62,70].

Beyond the acute window, repeated ketamine exposure in diabetes should prompt surveillance for urologic, hepatic, and cognitive adverse effects. A structured urinary history at baseline and follow-up, screening for frequency, urgency, dysuria, hematuria, or pelvic pain, with a low threshold for urinalysis and urology referral, is prudent, given the risk of ketamine-associated cystitis and obstructive uropathy in a population already vulnerable to nephropathy and autonomic bladder dysfunction [37,38]. Periodic liver enzyme monitoring may also be warranted during extended treatment courses, particularly among patients with non-alcoholic fatty liver disease or polypharmacy involving hepatotoxic agents [71,72]. Ongoing cognitive and mood reassessment can help identify patients in whom cumulative neurocognitive burden begins to outweigh therapeutic benefit.

Given persistent PK–PD uncertainties in diabetes, rigid fixed-dose regimens are not advisable. A “start low, go slow” approach is therefore appropriate, initiating at the lower end of standard subanesthetic dosing ranges with gradual titration according to clinical response and tolerability [35]. Concomitant use of strong CYP3A4 or CYP2B6 inhibitors or inducers, as well as antidiabetic agents that may modulate ketamine’s inflammatory and metabolic signaling (e.g., metformin, GLP-1 receptor agonists, insulin), should prompt heightened vigilance for changes in both efficacy and adverse effects, favoring cautious dose adjustments rather than empiric escalation [12,24,26,33]. Importantly, these recommendations represent interim clinical guidance; definitive dosing and monitoring parameters will require purpose-built trials in diabetic cohorts.

### 4.6. Limitations

English-language restriction and three databases may have missed evidence. The gray literature and unpublished data were excluded. Designs were heterogeneous (case reports, animal experiments, narrative reviews), precluding quantitative synthesis and limiting certainty. No formal risk-of-bias appraisal was conducted (scoping design). Most human data consists of single case reports or small, uncontrolled series, with limited follow-up and heterogeneous outcome measures; the resulting risk of selection and reporting bias is high. Preclinical models are also diverse in design and dosing. Therefore, findings should be viewed as hypothesis-generating rather than definitive.

## 5. Conclusions

Ketamine shows promise for TRD and DN in diabetes, with rapid effects that could indirectly improve self-management. Yet diabetes may alter exposure and glycemic risk, and long-term safety is insufficiently defined. We therefore recommend restricting use to carefully selected cases in multidisciplinary settings, with systematic monitoring and DDI review. Purpose-built popPK/PK-PD studies in diabetic cohorts should now define variability, interactions, and exposure–response to enable model-informed precision dosing.

Ketamine’s rapid antidepressant and analgesic effects are especially relevant for patients in whom conventional therapies have proven inadequate. By addressing both psychiatric and pain-related comorbidities, ketamine may indirectly improve glycemic control and overall quality of life, offering a novel avenue for integrated diabetes care. However, ketamine’s clinical utility in this population remains constrained by several limitations. Its pharmacology is complex, with biphasic effects on glucose metabolism (low doses associated with transient hyperglycemia and higher doses with hypoglycemia) that complicate metabolic management. Additional concerns include interactions with antidiabetic medications, hepatic enzyme modulation, cardiovascular and renal toxicity, and the potential for dissociative side effects or misuse. These risks are particularly concerning in individuals with diabetes, who already carry a high burden of metabolic, cardiovascular, and cognitive vulnerability.

The current evidence base is also limited. Most studies are small, short-term, and heterogeneous in design, with few trials explicitly including diabetic participants or monitoring diabetes-specific outcomes such as insulin sensitivity, HbA1c, or long-term microvascular complications. As such, routine use of ketamine in diabetes care is premature. Future research should prioritize large-scale, longitudinal clinical trials that incorporate PK/PD modeling in diabetic populations, evaluate interactions with antidiabetic therapies, and monitor both metabolic and psychiatric outcomes over time. Biomarker-guided approaches, exploration of enantiomer-specific effects (e.g., (R)-ketamine, HNK), and integration of pharmacogenomic data may help refine patient selection and dosing strategies.

Until such evidence is available, ketamine should not be considered a frontline therapy in diabetes care. Instead, its use should be restricted to carefully selected patients with refractory depression or neuropathic pain, administered within interdisciplinary care frameworks that include endocrinology, psychiatry, pain medicine, and primary care. This cautious, collaborative approach will help balance ketamine’s therapeutic potential against its substantial risks, while laying the groundwork for its possible future integration into precision diabetes management.

## 6. Future Perspectives

Research on ketamine in diabetes remains nascent, and several areas warrant further development. First, diabetes-specific popPK/PK-PD studies are essential to quantify how glycemic control, inflammation, renal function, and antidiabetic therapies influence ketamine exposure and clinical response. Such studies should incorporate chiral assays, metabolite profiling, and sparse sampling strategies suitable for real-world infusion and intranasal clinics, with rigorous time-stamping to strengthen exposure–response inference. Second, mechanistic work is needed to clarify ketamine’s bidirectional effects on glucose regulation, including contributions from autonomic activation, hepatic glucose output, and insulin dynamics. Understanding these pathways will be critical for preventing glycemic instability in vulnerable patients and may support biomarker-guided, tailored protocols. Third, interactions with first-line antidiabetic therapies, including metformin, GLP-1 receptor agonists, SGLT2 inhibitors, and insulin, should be systematically evaluated in both preclinical and clinical contexts to identify potential PK/PD conflicts that may alter efficacy, safety, or day-to-day diabetes management during induction and maintenance. Fourth, future RCTs should deliberately include diabetic populations and stratify participants by diabetes type, HbA1c, eGFR, and inflammatory biomarkers. Trials should capture both psychiatric and metabolic outcomes and define maintenance, tapering, and discontinuation strategies. In this context, distinguishing pharmacokinetic and metabolic risk profiles between induction, maintenance, and booster dosing phases represents an important unmet need, as cumulative exposure and inter-occasion variability may differentially affect glycemic stability and safety over time. Long-term follow-up is needed to determine whether ketamine meaningfully affects diabetes-relevant microvascular complications, cognitive trajectories, or cardiometabolic risk [67]. Lastly, equitable implementation strategies, including insurance coverage pathways, standardized monitoring, interdisciplinary care, culturally responsive education, and informed consent materials tailored to health literacy, will be essential for ensuring ketamine’s potential benefit reaches populations who disproportionately bear the burden of diabetes and depression. In parallel, real-world data from ketamine clinics and integrated health systems could complement trial evidence by capturing rare metabolic events, drug–drug interactions, and long-term safety signals not detectable in short-duration trials [73]. Collectively, advancing these research domains will be critical for defining ketamine’s therapeutic role in diabetes care and for developing precision-informed approaches that maximize benefit while minimizing risk.

## Figures and Tables

**Table 1:** Summary of included studies evaluating ketamine in diabetes (human and animal evidence). Primary studies are listed first, followed by secondary reviews. Doses and routes are standardized where reported.

Study	Design	Diabetes Context	Population/Model	Indication	Ketamine Regimen	Comparator	Outcomes	AEs	Main Findings
Lortrakul & Pattanaseri, 2024 [16]	Case report (primary)	T1DM	36-year-old male with TRD	Depression	Racemic IV 0.5 mg/kg over 40 min per session; 11 sessions/year	No comparator	Depressive symptoms; point-of-care glucose	Hypoglycemia (<70 mg/dL) in 4/11 sessions; dizziness, sweating, palpitations	Mood improved; drop in glucose after some infusions—recommend stringent monitoring
Easterly & Taylor, 2023 [17]	Case report (primary)	T1DM (adolescent)	Psychiatric anesthesia setting	Depression/SI	Ketamine–propofol; multiple anesthetics (doses not reported)	No comparator	PROMIS depression/SD measures	Subjective memory complaints	Marked depressive symptom improvement; SI resolved
Alzahid et al., 2024 [18]	Case report (primary)	T1DM (adolescent)	Orthopedic reduction	Analgesia/sedation	Single-episode ketamine sedation (dose NR) + morphine	No comparator	Pain relief; reduction success	NR	Successful closed reduction; highlights multimodal pain needs in T1DM
Harris et al., 2024 [19]	Case report (primary)	T2DM	29-year-old male (bipolar, ASD)	KAP for mood/behavior	6 IV infusions titrated over 1 month (40–90 mg) + 2 boosters (90–110 mg) + psychotherapy	No comparator	GAD-7, PHQ-9, C-SSRS, PCL-5	NR	Reduced outbursts and symptom scores; no reported metabolic AEs
Han et al., 2023 [20]	RCT (primary)	GDM, post-cesarean	n = 140	Post-op analgesia	Esketamine 0.5 mg/kg in IV PCA with sufentanil + ondansetron up to 48 h	Placebo PCA	Sufentanil use; VAS pain at 6/24/48 h; bowel function	Nausea/vomiting/dizziness similar between groups	Less opioid use; lower movement-evoked pain; faster bowel recovery
Rastogi & Jude, 2021 [21]	Narrative review (secondary)	DN (T1DM/T2DM)	Multiple small trials	Neuropathic pain	Topical ketamine 5% (±amitriptyline)	No comparator	Pain VAS; neuropathic symptoms	Minimal (local irritation)	Mixed results; adjunctive role suggested
Elbeddini et al., 2024 [22]	Narrative review (secondary)	DN	Multiple studies	Neuropathic pain	Topical ketamine 2% (often with amitriptyline 4%)	Placebo and oral gabapentin	Pain; sleep interference; onset	Local burning/redness/rash	Moderate pain relief; favorable tolerability vs. oral gabapentinoids
Yilmaz & Dokuyucu, 2023 [23]	Animal (primary)	STZ-T1DM	Wistar rats	Anesthesia dynamics	Ketamine 80 mg/kg i.p. + xylazine 12 mg/kg i.p.	Non-diabetic controls	Induction time; weight; glucose	NR	Faster induction in diabetic rats; weight lower; glucose higher
Sedky & Magdy, 2021 [24]	Animal (primary)	Diet + low-dose STZ T2DM	Wistar rats	Cognition/behavior	Ketamine 25 mg/kg/day i.p. ×7 days; liraglutide 300 µg/kg/day s.c. ×4 weeks	Multiple groups	Open field; water maze; TNF-α, MDA, GSH, BDNF; histology	NR	Diabetes worsened ketamine-related hyperlocomotion/cognitive deficits; liraglutide attenuated inflammation/behavioral changes
Chavda & Patel, 2022 [25]	Animal (primary)	T2DM + MCAo stroke	Wistar rats	Neurocognition	Ketamine 100 mg/kg i.p. + xylazine 10 mg/kg i.p., daily ×7 days pre/post	Antidiabetic therapy arms	Maze latency; oxidative stress; neurochemistry	NR	Chronic anesthesia worsened cognition/stress markers; voglibose/saxagliptin improved outcomes
Tao et al., 2023 [26]	Animal (primary)	T2DM + cardiac I/R	SD rats	Cardiac injury/inflammation	Ketamine 1 mg/kg i.v. ± insulin ×10 days	Controls	Glucose; AST/LDH/CK-MB; IL-1β/IL-6/TNF-α; autophagy	NR	Ketamine ± insulin reduced injury and inflammation combination superior

Abbreviations: AEs, adverse events; ASD, autism spectrum disorder; AST, aspartate aminotransferase; BDNF, brain-derived neurotrophic factor; C-SSRS, Columbia-suicide severity rating scale; CK-MB, creatine kinase-MB isoenzyme; DN, diabetic neuropathy; GAD-7, generalized anxiety disorder-7 scale; GDM, gestational diabetes mellitus; GSH, glutathione; I/R, ischemia/reperfusion; IL-1β, interleukin-1β; IL-6, interleukin-6; i.p., intraperitoneal; IV, intravenous; KAP, ketamine-assisted psychotherapy; LDH, lactate dehydrogenase; MCAo, middle cerebral artery occlusion; MDA, malondialdehyde; NR, not reported; PCA, patient-controlled analgesia; PCL-5, post-traumatic stress disorder checklist for DSM-5; PHQ-9, patient health questionnaire-9 (depression severity scale); PROMIS, patient-reported outcomes measurement information system; RCT, randomized controlled trial; s.c., subcutaneous; SD, Sprague Dawley; SI, suicidal ideation; STZ, streptozotocin; T1DM, type 1 diabetes mellitus; T2DM, type 2 diabetes mellitus; TNF-α, tumor necrosis factor-α; TRD, treatment-resistant depression; VAS, visual analogue scale.

**Table 2:** Selected pharmacokinetic and pharmacodynamic drug–drug interactions relevant to ketamine administration in patients with diabetes mellitus. The table summarizes metabolic pathways, expected effects on ketamine or its active metabolites, and diabetes-specific clinical considerations, including glycemic variability, neurocognitive effects, and risk of ketoacidosis. Interactions are based on mechanistic, preclinical, and limited clinical evidence and should be interpreted in the context of dose, route of administration, and individual metabolic status.

Drug/Class	Mechanisms Relevant to Ketamine	Expected Effect on Ketamine/Metabolites	Potential Diabetes-Specific Clinical Implications
Strong CYP3A4 inhibitors (e.g., azoles, macrolides) [27,33]	Inhibit N-demethylation and hydroxylation	↑ ketamine/norketamine exposure	Greater dissociation, hemodynamic effects; consider lower starting dose and closer monitoring
Strong CYP3A4/2B6 inducers (e.g., some anticonvulsants, rifampin) [27,33]	↑ metabolic clearance	↓ exposure, shorter duration of effect	Reduced antidepressant/analgesic benefit; may require higher or more frequent dosing
Metformin [27,58]	Alters hepatic gluconeogenesis, may interact with AMPK/mTOR signaling	Possible attenuation of sustained antidepressant-like effects	Monitor depressive response and glycemic control; consider alternative antidepressant strategies if response is blunted
GLP-1 receptor agonists [24]	Improve insulin sensitivity; may modulate neuroinflammation	Potentially synergistic effects on inflammation and mood	Unknown PK interaction; monitor glucose and GI AE’s
SGLT2 inhibitors [12,59,60]	Promote glucosuria, risk of euglycemic DKA	No direct CYP interaction; osmotic diuresis may interact with ketamine-related hemodynamic shifts	Monitor ketones and volume status in those with high DKA risk
Insulin/insulin secretagogues [12,13]	Major determinant of hypoglycemia risk	No direct CYP effect; alters background glucose dynamics	Adjust insulin doses around infusion sessions; frequent bedside glucose checks

↑ and ↓ denote increased and decreased ketamine or metabolite exposure/effect, respectively.

## Data Availability

No new data were created or analyzed in this study.

## Abbreviations

The following abbreviations are used in this manuscript:

ADA	American Diabetes Association
AEs	Adverse events
AMPA	α-amino-3-hydroxy-5-methyl-4-isoxazolepropionic acid
ALT	Alanine Aminotransferase
AST	Aspartate Aminotransferase
AUC	Area Under Curve
BDNF	Brain-Derived Neurotrophic Factor
BLQ	Below-limit-of-quantification
BMI	Body mass index
BP	Blood pressure
BSV	Between-subject variability
CKD	Chronic Kidney Disease
CK-MB	Creatine Kinase–Myocardial Band
CL	Clearance
C_max	Maximum Concentration
CNS	Central Nervous System
COMT	Catechol-O-Methyltransferase
CRP	C-reactive protein
CVD	Cardiovascular disease
CYP2B6	Cytochrome P450 Family 2 Subfamily B Member 6
CYP2C9	Cytochrome P450 Family 2 Subfamily C Member 9
CYP3A4	Cytochrome P450 Family 3 Subfamily A Member 4
CYP450	Cytochrome P450
DDI	Drug–drug interaction
DHNK	Dehydroxynorketamine
DN	Diabetic Neuropathy
eGFR	Estimated Glomerular Filtration Rate
E_max	Maximum effect
FDA	Food and Drug Administration
FOCEI	First-Order Conditional Estimation with Interaction
GABA	Gamma-Aminobutyric Acid
GDM	Gestational diabetes
GLP-1	Glucagon-Like Peptide-1
HbA1c	Hemoglobin A1c
HCN	Hyperpolarization-activated cyclic nucleotide-gated
HDRS	Hamilton Depression Rating Scale
HNK	Hydroxynorketamine
HPA	Hypothalamic–Pituitary–Adrenal (axis)
HR	Heart Rate
IL-1β	Interleukin-1 beta
IL-6	Interleukin-6
IM	Intramuscular
IN	Intranasal
IOV	Inter-occasion variability
i.p.	Intraperitoneal
I/R	Ischemia/Reperfusion
IV	Intravenous
KAP	Ketamine-Assisted Psychotherapy
KET	Ketamine
LDH	Lactate Dehydrogenase
LFT	Liver Function Tests
MADRS	Montgomery–Åsberg Depression Rating Scale
MCAo	Middle Cerebral Artery Occlusion
MDD	Major Depressive Disorder
MIPD	Model-informed precision dosing
mTor	Mammalian target of rapamycin
NMDA	N-Methyl-D-Aspartate
norKET	Norketamine
PCP	Phencyclidine
pcVPC	Prediction-Corrected Visual Predictive Check
PK	Pharmacokinetic
PD	Pharmacodynamic
PK-PD	Pharmacokinetic-pharmacodynamic
PO	Per os (by mouth)
popPK	Population pharmacokinetics
PRISMA	Preferred Reporting Items for Systematic Reviews and Meta-Analyses
RCT	Randomized controlled trial
SAEM	Stochastic Approximation Expectation-Maximization
SBP	Systolic Blood Pressure
s.c.	Subcutaneous
SGLT2	Sodium-Glucose Co-Transporter 2
SNRI	Serotonin-Norepinephrine Reuptake Inhibitor
SSRI	Selective Serotonin Reuptake Inhibitor
T1DM	Type 1 Diabetes Mellitus
T2DM	Type 2 Diabetes Mellitus
TCA	Tricyclic Antidepressant
T_max	Time to maximum concentration
TNF-α	Tumor Necrosis Factor-alpha
TRD	Treatment-Resistant Depression
VAS	Visual Analog Scale for pain
VPC	Visual Predictive Check
WHO	World Health Organization
